# Multigene tests for breast cancer: the physician’s perspective

**DOI:** 10.18632/oncotarget.27948

**Published:** 2021-04-27

**Authors:** Francesco Cognetti, Laura Biganzoli, Sabino De Placido, Lucia del Mastro, Riccardo Masetti, Giuseppe Naso, Giancarlo Pruneri, Donatella Santini, Carlo Alberto Tondini, Corrado Tinterri, Giuseppe Tonini, Sandro Barni

**Affiliations:** ^1^Scuola di specializzazione di Oncologia, La Sapienza University, Rome, Italy; ^2^Sandro Pitigliani Medical Oncology Department, Hospital of Prato, Prato, Italy; ^3^Università Degli Studi di Napoli Federico II Dipartimento di Medicina clinica e Chirurgia Professore di Oncologia Medica, Napoli, Italy; ^4^Oncology, IRCCS AOU San Martino - IST Istituto Nazionale per la Ricerca del Cancro, Genova, Italy; ^5^Breast Center, Catholic University, Rome, Italy; ^6^Department of Radiology, Pathology and Oncology, La Sapienza University, Rome, Italy; ^7^Department of Pathology, Fondazione IRCCS Istituto Nazionale Tumori, Milan, Italy; ^8^Department of Pathology, IRCCS Azienda Ospedaliera Universitaria di Bologna, Policlinico di Sant’Orsola, Bologna, Italy; ^9^Medical Oncology, Papa Giovanni XXIII Hospital, Bergamo, Italy; ^10^Senology Unit, Humanitas Cancer Center, Rozzano, Milan, Italy; ^11^Medical Oncology, School University Campus Bio-Medico, Rome, Italy; ^12^Emeritus, Department of Oncology, ASST Bergamo Ovest, Treviglio, Bergamo, Italy

**Keywords:** genomic tests, breast cancer, adjuvant chemotherapy, adjuvant hormone therapy

## Abstract

Breast cancer is the most common tumour in women and the first cause of death for cancer in the female population. Preserving the quality of life has therefore become an important objective in the management of the disease. The benefits of adjuvant chemotherapy in patients with HR+ HER2- early breast cancer should always be balanced against its potential short and long-term adverse effects, and identifying the appropriate patients for whom chemotherapy can offer the highest clinical benefit is critical. Besides clinical and pathological factors, today four multigene tests able to guide the choice of the adjuvant therapy early breast cancer are available in Italy: Oncotype DX^®^, EndoPredict^®^, MammaPrint^®^ e Prosigna^®^. This review evaluates the main characteristics of these diagnostic tests, the studies on clinical utility, their economic impact and their inclusion in international and national guidelines. The Oncotype DX Breast Recurrence Score^®^ test is the only multigene test validated, with level IA evidence, to guide the adjuvant therapy decisions: hormone therapy alone for most patients with RS results 0–25, and chemotherapy for patients with RS results 26–100. Clinical data demonstrate that the Oncotype DX test is able to significantly impact therapeutic decisions, reducing chemotherapy use up to 49% and supporting the use of chemotherapy (up to 12%) in potentially under-treated patients. Based on the level of clinical evidence and established clinical utility, several multigene tests have been included in the main international guidelines, with recommendations ranging from “strong” to “moderate”.

## INTRODUCTION

Breast cancer is the most common cancer in women and the first cause of death for cancer in the female population [1]. Data 2015 by the World Health Organization (WHO) indicate an estimated incidence for Europe of 95 cases per 100,000 people, with a mortality rate of 23/100,000 [2]. In Italy, the new cases of breast cancer in 2019 were 53,000 [1, 3].

Breast cancer is responsible for 30% of malignant tumors in women, with a higher incidence in the younger population: 40% in women less than 50 years of age; 35% between 50 and 69 years and 22% in women over 70 years. The trend of new diagnoses is slightly increasing (+ 0.3%), due to the early screening and the decreased mortality (–0.8%), especially in younger age groups (–0.9%). As far as prevalence is concerned, in Italy about 800,000 women have a diagnosis of breast cancer: 44% of the global female population diagnosed with cancer and 24% of all prevalent cases [3]. About two/third of women with breast cancer have a positive-hormone receptor disease [4].

The number of deaths, certified by the Istituto Nazionale di Statistica (ISTAT) in 2016, was 12,000; this figure places breast cancer in first place among the causes of death for cancer in the Italian female population, regardless of age groups. The spread of the early screening significantly improved the prognosis of the disease [5, 6] and at the time being, 80% of patients diagnosed with early breast cancer have a survival > 10 years. Preserving the quality of life has therefore become an important objective in the management of the disease. To date, the burden of the disease is significant, this is also due to the side effects experienced during and after adjuvant therapies (radio-, chemo- and hormone therapy).

## DIAGNOSIS AND TREATMENT OF BREAST CANCER

The diagnosis of breast cancer is based on clinical evaluation, imaging and histopathological analysis of the tumor tissue, the latter also provides information on important prognostic factors. The main independent prognostic factors are: tumor size, lymph node status, histological grade [7, 8]. The expression of Ki67, the receptors for estrogen and progesterone (ER and PR), of HER2 (human epidermal growth factor receptor 2) and related proteins are also evaluated by immunohistochemistry. ER/PR and HER2 expression are both prognostic and predictive of the response to hormonal and anti-HER2 therapy respectively.

The treatment of early breast cancer is mainly based on locoregional surgery with or without radiotherapy. The risk of distant relapses (after 5 years) is continuous, especially in case of ER positive/HER2 negative cancers, which alone represent almost half of distant relapses [4]. To date, it is unclear whether an adjuvant chemotherapy is necessary for all these cases in order to effectively prevent these situations. An overview carried out by the Early Breast Cancer Trialists’ Collaborative Group (EBCTCG) in 2012 showed that only about 10% of patients benefit from chemotherapy, but none of the classical clinical or pathological parameters allowed to identify any of these patients [9].

### Adjuvant chemotherapy in the management of early breast cancer

Cytotoxic agents inhibit molecular mechanisms responsible for cell proliferation. Therefore, the chemo-sensitivity strongly depends on the biology of the tumor. In patients with HR+ cancers, the analysis and the identification of the tumour gene profile is important to better predict the benefits of chemotherapy and to guide treatment decision [10]. The importance of carefully evaluating the toxicity of chemotherapy with respect to its potential benefits is linked to the possible occurrence of acute and “late” side effects arising during treatment; in some cases they can resolve within a few months of its conclusion, but many times they do not resolve, such as in case of anthracycline cardiotoxicity [11, 12].

### Impact of treatment on patients’ quality of life

The consequences of both disease and treatments have a significant impact on the patient’s well-being, as well as indirect implications for society, when patients have to stop work for long periods of time and bear the high costs of the treatment, which can lead to new morbidity and disability. The toxicity of chemotherapy can be particularly high, both in the short term (nausea, vomiting, anorexia, diarrhea, marrow deficit, cystitis, amenorrhea, neurotoxicity are the most commonly reported adverse events) and in the medium - long term [13]. In the latter case, adverse events are fortunately not frequent but the severity of the events is generally high. The most important events are cardiotoxicity (in particular caused by anthracycline and trastuzumab) and haematological toxicity. Cardiotoxicity can also occur very late in life. Chemotherapy can lead to a very disabling temporary or permanent condition, which have negative impact on the quality of life and consequent limitations of daily activities (work and recreation) not only of patients but also of their care givers [14, 15]. The choice of appropriate adjuvant therapy or a correct combination of adjuvant therapies is a particularly difficult challenge for clinicians. Indeed, the decision can be based on algorithms often not very applicable in clinical practice (designed on populations not representative of real-life) and/or on traditional clinical-pathological parameters, that proved to be poor in identifying patients for whom chemotherapy could be spared. Unfortunately, this often results in over-treatment and under-treatment, with short and long term consequences on the patient’s life; another added factor is the lack of an homogeneous approach to chemotherapy from both a number of cycles and dosages/types of drugs used point of view.

## MULTIGENE TESTS SUPPORTING CLINICAL DECISIONS

As already discussed, treatment decision for patients with breast cancer is based on prognostic clinical-pathological parameters (e.g., age, tumor size, presence of node metastases and histological grade), in addition to predictive factors for response to the treatment (ER/PgR and HER2). The combination of these factors in decision algorithms can support clinicians in the choice of the treatment options.

Nowadays, multigene tests able to guide the choice of the adjuvant therapy are available [16]. The expected benefit of multigene tests, when combined with clinical-pathological prognostic indicators, is to provide additional prognostic information (typically expressed in risk levels or score) in terms of clinical outcomes (development of metastases at 10 years) [17]. The use of multigene tests can result in an optimal patient selection and could avoid the toxic effects of adjuvant chemotherapy in patients who would not really benefit from it.

Four multigene tests for early breast cancer are available in Italy: *Oncotype DX*^®^, *EndoPredict*^®^, *MammaPrint*^®^ and *Prosigna*^®^, validated in clinical and pharmacoeconomic trials. The marketed tests are not equivalent; they have been designed to evaluate the expression of different genes and to solve different diagnostic issues. For this reason, it is not surprising that the results obtained by the various tests are not overlapping and have a relatively low concordance.

The available tests evaluate different genes with different objectives and can be classified according to their prognostic and predictive values for chemotherapy, their ability to analyze molecular subtypes, their availability (local or centralized) or on the supporting clinical evidence. One of the most important aspect of these tests is the ability to provide an estimate of the relapse risk with the hormone therapy alone (prognostic value), and to predict the benefit from chemotherapy (predictive value). The prognostic value is associated with the natural history of the disease and defines the risk of an event (e.g., distant relapse) regardless of the administered treatment.

A biomarker is considered predictive if it is associated with a clinical outcome in a treatment-dependent manner. The predictive value of a test relates to its ability to provide information on the susceptibility and resistance to a specific therapy [10]. A positive predictive value (PPV) is associated with a benefit from the treatment and a negative predictive value (NPV) is associated with an absent or unfavorable outcome obtained with a specific therapy.

## CHARACTERISTICS OF THE MULTIGENE TESTS AVAILABLE IN ITALY

As mentioned, the available multigene tests provide different information and therefore are not interchangeable. Therefore, the choice of a test for the single patient should be made according to the question to be addressed and the characteristics of the different multigene assays. These differencies rely on the gene selection on the patient population used to validate the assays and importantly on their demonstrated clinical utility. The four tests available in Italy are described below.

### Oncotype DX breast recurrence score test^®^

- The Oncotype DX test provides two key informations: the risk of relapse of the breast cancer within 10 years (prognostic value) and the estimated outcome if treated with adjuvant chemotherapy (predictive value). The test validation included prospective randomized clinical trials elevating the level of evidence to IA.
- Description of the technology. The Oncotype DX test uses qRT-PCR technology to analyze the expression level of 21 genes on a Formalin-Fixed Paraffin-Embedded (FFPE) sample. A clinically validated algorithm, calculates an individual score on a scale from 0 to 100, based on the gene expression results. The score, expressed as a Recurrence Score^®^ result, estimates the relapse risk within 10 years and the probability of response to chemotherapy. To date, the Oncotype DX test is available in a centralized lab: the hospital receives the sample preparation kit which is then sent to the manufacturer’s central laboratory (California, USA) for the analysis.

### MammaPrint^®^ (since 2018: MammaPrint^®^ & BluePrint^®^)

- This test classifies the molecular subtype of the tumour and estimates the risk of relapse within 10 years for untreated patients and patients treated with hormone therapy alone. The test prognostic value was clinically validated in a prospective randomized trial [18, 19].
- Description of the technology. The test, based on microarray-RNA technology, analyzes the expression profile of 70 genes on a FFPE sample. The test provides a numerical index (MP index) with values ranging from –1 to +1. This value is related to a binary system of clinical risk classification (high or low). The test is performed in 2 central labs (Amsterdam – the Netherlands and California) which collect and analyze the samples. Recently, the manufacturer also provided a kit to perform the test locally, but this kit needs the local availability of an NGS sequencer and the necessary expertise for the preparation of the samples.

### EndoPredict^®^

- EndoPredict^®^ is a prognostic test that estimates the risk of relapse within 10 years and provides information on possible long-term hormone therapies (after 5 years). The test is available in Italy since 2014.
- Description of the technology. The qRT-PCR technique evaluates the expression level of 12 genes on a FFPE sample [20–23]. The molecular score of this test is then combined with the clinical characteristics of the tumor (size and lymph nodes status, providing a risk score, named the EPclin, estimatingthe 10-years rate of recurrence (with 5 years of hormone therapy alone). The test can be supplied as kit, if the hospital is equipped, otherwise it can be performed in a centralized laboratory in Salt Lake City (Utah, USA).

### Prosigna^®^

- Prosigna^®^ is a prognostic test able to estimate the 10 years risk of relapse. This test is also used for research purposes to analyze the molecular subtypes of the tumour.
- Description of the technology. The test evaluates the the expression of 50 genes (PAM50) on a FFPE sample. The RNA extracted from the sample is processed through the nCounter Dx Analysis System (NanoString Technologies). The Prosigna^®^ genomic score PAM50 defines a tumor subtype (Luminal A/B, Basal/like, or HER2-), and the Risk Of Recurrence (ROR) index, a value ranging from 0 to 100, is calculated by combining the genomic score analysis with a proliferation index and the size of the tumor.. The test is available as a diagnostic kit to be performed on site in equipped labs.

Table 1 compares the main characteristics of the 4 tests.

**Table 1:** Multigene test for identifying the gene expression of the early breast cancer

	Oncotype DX^®^	MammaPrint^®^&BluePrint^®^	EndoPredict^®^	Prosigna^®^
**Service/Kit**	Service of centralized test^**^	Service of centralized test* Kit test for local use	Service of centralized test^***^ Kit test for local use	Kit test for local use
**Objective**	Relapse risk (prognostic value) at 9 years patients treated with hormone therapy only and benefit from chemotherapy (predictive value).	Relapse risk at 5 years and benefit from chemotherapy (molecular subtype)	Relapse risk at 10 years from diagnosis in patients treated with hormone therapy. Information on long-term hormone therapy (>5 years).	Relapse risk at 10 years from diagnosis in patients treated with hormone therapy only and molecular subtype
**Number of analysed genes**	21 genes *(16 tumours-related and 5 reference genes)*	70 genes (MammaPrint) 80 genes (BluePrint) *Related to several aspects of tumour biology*	12 genes *genes 8 tumours-related genes, 3 normalization genes and 1 control gene*	58 genes *50 geni for the identification of the molecular subtype and 8 control genes*
**Analytical test**	qRT-PCR	NGS	qRT-PCR	Direct hybridization
**Tissue sample**	Formalin fixed and paraffine embedded (FFPE) tumour tissue	Formalin fixed and paraffine embedded (FFPE) tumour tissue	Formalin fixed and paraffine embedded (FFPE) tumour tissue	Formalin fixed and paraffine embedded (FFPE) tumour tissue
**Patients characteristics**	Patients with ER+ HER2- early breast cancer, without nodes involvement (N-) or maximum 3 involved nodes (N1)	Pre- or post-menopausal women with stage I/II breast cancer, tumour volume ≤ 5 cm, ER+/ER-, without nodes involvement (N-) or maximum 3 involved nodes (N1)	Patients with ER+ HER2- early breast cancer, without nodes involvement (N-) or maximum 3 involved nodes (N1) during hormone adjuvant therapy	Post-menopausal women with ER+/PgR+, HER2- breast cancer, without nodes involvement (stage I or II) or maximum 3 involved nodes (stage II or IIIa)
**Test results**	Continuous value Recurrence Score results	Continuous value (plus tumour subtype) MP index	Continuous value Molecular score (based on level of gene expression) EPclin score (a combination of molecular score and tumour volume and nodes status)	Continuous value Molecular subtype Risk of Recurrence (ROR) Score (a combination of genetic and clinical data)
**Classification of relapse risk**	Different intervals and risk cut-offs based on nodes involvement and patient’s age	Low risk^****^ High risk^****^	EPclin = 3,3 (cut-off for high and low risk)	*Any node involved:* 0–40 = Low Risk 41–60 = Intermediate Risk 61–100 = High Risk *1–3 nodes:* 0–40 = Low Risk 41–100 = High Risk

^*^Two labs: one in Europe (Amsterdam) e and the other in USA (Irvine, California). ^**^One lab in USA (Redwood City, California). ^***^One lab in Salt Lake City (Utah, USA). ^****^The Manufacturer states that in case of MPI index values near to cut-off (± 0.050), the accuracy of the classification is < 90%. Abbreviations: ER, estrogen receptor; FFPE, formalin fixed and paraffine embedded; HER2, human epidermal growth factor receptor 2; NGS, Next Generation Sequencing; PgR, progesterone receptor; qRT-PCT, quantitative reverse transcription polymerase chain reaction.

## STUDIES ON CLINICAL UTILITY AND ECONOMIC IMPACT

To be used in the clinical practice, a test need to be more precise and accurate than conventional parameters in guiding decisions regarding the use of adjuvant chemotherapy [24]. Table 2 shows the number and characteristics of clinical and economic trials of the 4 tests.

**Table 2:** Characteristics of the different clinical and economic trials for the 4 multigene tests and score for the early breast cancer

	Oncotype DX test	MammaPrint	EndoPredict (EP)	PAM50/Prosigna
Clinical validation	20 trials: A(2) B(8) D(10)	21 trials: A(1) C(3) D(17)	4 trials B(4)	5 trials B(5)
Clinical usefulness	22 trials	4 trials	1 trial	1 trial
Economic evaluation	32 evaluations	7 evaluations	1 evaluation	–

(A) prospective trial; (B) prospective trial on file samples; (C) prospective real-life trial; (D) retrospective real-life trial [24].

## RANDOMIZED CLINICAL TRIALS

Table 3 shows the results of the 4 published randomized controlled trials (RCT; TAILORx, NCT00310180; MINDACT, NCT00433589; RxPONDER, NCT01272037, PLAN B, NCT01049425). The trials compare the use of multigene tests with the clinical practice, evaluating the adjuvant chemotherapy in patients with early breast cancer (stage I, II or III operable) with 0–3 positives nodes, ER+ (and/or PgR-positive) and HER2-.

**Table 3:** Characteristics and outcomes of phase III randomized clinica trials on the clinical use of multigene tests approved in Italy

Trial	Enrolled population	Primary endpoint	Secondary endpoints
**TAILORx** **[27, 28, 29]** **(f-up: 9 years)**	*N* = 10273; ER+ and/or PgR+), HER2- early breast cancer (stage IA-IIIB, LN 0-3) with positive nodes, Treatment: hormone vs chemo-hormone therapy	**Disease-free survival:** HR 1.08 (95% CI, 0.94–1.24; *P* = 0.26) Disease-free survival at 9 years: 83.3% and 84.3% The benefit from CT ranged with combination of RS and age (*P* = 0.004), The benefit from CT in women ≤ 50 years with a 16–25 RS	**No distant relapses** (94.5% vs 95.0%) **Absence of distant or locoregional relapses** (92.9% vs 92,9%) **Overall survival** (93.9 vs 93.8)
**MINDACT [19]** **(f-up: 5 years)**	*N* = 6693; early breast cancer (stage T1, T2 or T3 operable; LN 0–3). Treatment: hormone vs chemo-hormone therapy	**Relapse-free survival rate at 5 years** 94.7% (95% CI: 92.5–96.2) for HT only. The difference for HT arm and HT + CT arm was 1,5 points (lower rate for HT only)	**Survival and disease-free survival in patients after classification of relapse risk (clinical criteria and 70 gene multigene test)** Not complete concordance
**RxPONDER** **(planned f-up: 15 years. ongoing: 2001–2022)**	ER+ HER2- early breast cancer, RS < 25 Treatment: hormone vs chemo-hormone therapy	**Interaction between RS and benefit from CT**	**Overall survival** **Distant disease-free survival (DFS)** **Local disease-free interval** **Toxicity according NCI-CTCAE standard, version 4.0**
**PLAN B [25, 26]**	HR-/HER2 LN 0-3 early breast cancer Treatment: hormone therapy vs different CT regimens (with or without antracyclines)	**Disease-free survival in patients with RS > 12 treated with a combination and with RS 0-11 treated with HT only** 94%	

Abbreviations: CT, chemotherapy; ER, estrogen receptor; f-up, follow-up; HER2, human epidermal growth factor receptor 2; HR, hazard ratio; HT, hormone therapy; CI, confidence interval; LN, lymph node; NCI-CTCAE, National Cancer Institute Common Terminology Criteria for Adverse. Events. Only Oncotype DX (3 trials) and Mappaprint (1 trial) test were evaluated in phase III randomized trials.

### PLAN B trial [25]

The prognostic value of the Oncotype DX test was confirmed by the phase III Plan B clinical trial, which evaluated 5-year survival of 3,198 patients with HR+, HER2– early breast cancer and negative and positive nodes. Patients with RS results 0–11 were treated with hormone therapy alone, while patients with RS results 12–25 were randomized to hormone therapy plus chemotherapy (with or without anthracyclines) [25, 26]. The rate disease-free survival of patients with RS results 0–11 treated with hormone therapy only was 94% both for N0 patients with high clinical risk and N1 patients.

### TAILORx trial [27–29]

The predictive value of the Oncotype DX test was validated in 10,273 node-negative patients enrolled in the prospective, randomized TAILORx (Trial Assessing Individualized Options for Treatment) clinical trial. The benefit of the test to assess value of chemotherapy was evaluated in patients with RS results 11–25 and with clinical-pathological characteristics that met the criteria for hormone-chemotherapy. The analysis showed the non-inferiority of chemotherapy added to to hormone therapy compared to hormone therapy alone (HR 1.08; 95% CI 0.94–1.24; *p* = 0.29), indicating that patients with RS results 11–25 overall do not benefit from chemotherapy and can be treated with hormone therapy alone (some benefits from chemotherapy were suggested by exploratory analyses in women aged ≤ 50 years with RS results of 16–25) [28]. Overall, these results show that the Oncotype DX test can identify patients (about 20%) who substantially benefit from chemotherapy and patients where chemotherapy can be avoided (about 80%).

### Rx PONDER trial (https://clinicaltrials.gov/ct2/show/record/NCT01272037)

This phase III randomized trial is ongoing. The objective of the study is refine chemotherapy benefit according to the Recurrence Score results for 5,000 patients with HR+ HER2- breast cancer and 1–3 positive nodes, and a RS result 0–25.

### MINDACT trial [19]

The potential prognostic value of the MammaPrint^®^ test was evaluated by the MINDACT trial in 6,693 women with operable pT1-2 or T3, N0 or N+ (up to 3 lymph nodes) breast cancer. The primary endpoint was the distant metastasis free survival, of high-clinical risk patients and a low MammaPrint score profile, treated without chemotherapy (*n* = 644). Among the secondary endpoints, the study showed that high-clinical risk and low-MammaPrint risk patients randomized to chemotherapy had limited additional therapeutic benefits from adjuvant chemotherapy.

## COMPARATIVE TRIALS

Trials comparing multigene tests showed that they are not interchangeable.

- TransATAC [30]. A retrospective analysis on 774 biopsy samples of post-menopausal women with ER+ HER2- breast cancer treated for 5 years with tamoxifen or anastrazole and enrolled in the ATAC study, compared the prognostic value of 6 tests: Oncotype DX, Prosigna, EndoPredict, Breast Cancer Index (BCI), Clinical Treatment Score and immunohistochemistry with 4-markers. This analysis revealed different prognostic value of the assays.
- OPTIMAPrelim trial [31]. The study was performed in 35 UK hospitals and evaluated the results of 3 multigene tests (Oncotype DX, MammaPrint and Prosigna) on patients > 40 years with HR+/HER2- early breast cancer and node involvement (1 to 9) or, alternatively, tumour size > 3 cm and N0. The results showed that when assessed on the same patient samples each test provides different risks estimates.
- An analysis on 38 trials, presented at the ASCO Congress 2018, compared the clinical utility of 4 multigene tests on the use of chemotherapy in patients with ER+ early breast cancer and node negative. The results of this analysis are reported in Table 4 [32].

**Table 4:** Comparative analysis of the outcomes of multigene test on chemotherapy use [32]

	No test	EndoPredict	MammaPrint	Oncotype DX test	Prosigna
CT use, %	51	56	64	31	49
DR over 10 years, *n*	271	259	269	241	273
ED + H over 10 years, *n*	2260	2274	2435	1630	2113
10-year total cost of care	$72.9 M	$95.1 M	$102.2 M	$67.5 M	$88.0 M

Abbreviations: CT: Chemotherapy; DR: distant relapse; ED + H: Emergence Department + Hospitalization.

## REAL-LIFE REGISTRIES

The real-life data from registries provide additional information to those of RCT.

### TRANSBIG registry

From 2004 to 2011, the registry enrolled patients with breast cancer. Biopsy samples of 307 patients ≤ 60 years with ER+ (71%) or ER- (29%) breast cancer who did not receive adjuvant therapy [33] were selected and analyzed with the MammaPrint test. 90% of low risk patients were metastases-free after 10 years, as well as 69% of high risk patients.

### SEER registry (Surveillance, Epidemiology, and End Results Registry)

An observational study on 80,605 patients with HR+, HER2– breast cancer and node-negative or node-positive (up to 3) confirmed that patients with low Recurrence Score results with the the Oncotype DX^®^ test have excellent clinical outcome when receiving hormone therapy alone [34].

### CLALIT registry

Data from the Clalit registry, the largest health organization in Israel which approved the reimbursement of the Oncotype DX multigene test for patients with HR+ breast cancer, were separately analyzed for node-negative and node-positive. The results from the 1801 N0 patients supports evidence from the prospective TAILORx trial [35, 36]. With regard to node-positive patients (*n* = 755), the results support the use of the endocrine therapy alone in patients with ER+ HER2– N1 breast cancer for patients with RS results 0–17.

### PONDx clinical-practice study

1,738 patients from 27 Italian centers in 6 regions (Lombardy, Lazio, Campania, Abruzzo, Marche and Emilia Romagna) were enrolled in this study. The objective was to define the impact of the Oncotype DX test on the decision to use chemotherapy and to identify patients who can really benefit from testing. The results confirmed that the Oncotype DX Breast Recurrence Score test substantially impacted treatment decisions: the test reduced chemotherapy use by up to 49% and supported the use of chemotherapy by up to 12%. Overall, a large reduction in the use of adjuvant chemotherapy was observed, despite it was recommended initially for only 31% of patients The re-evaluation of PONDx results in light of the publication of TAILORx showed that the treatment with hormone therapy alone could be possible in 75% of cases. Such treatment changes could potentially lead to saving for the health care system and increase the quality of life of patients [37].

## EVALUATION OF THE ECONOMIC IMPACT OF MULTIGENE TESTS

The availability of multigene tests and the supporting results from clinical practice and randomized trials highlight the importance of their use to support and complement the traditional clinical parameters. However, the cost of these assays is not irrelevant: for this reason, it is essential to make an accurate assessment of the clinical situations where their use is most appropriate [38–41].

A recent systematic review [24] analyzed four criteria related to the evaluation of the value to be attributed to MammaPrint^®^, Oncotype DX^®^, Prosigna^®^ and EndoPredict^®^ genomic tests: methodology and test development, clinical validation, clinical utility and the economic value of multigene prognostic tests. The review by Blok et al. is based on 44 studies, most of them (32 studies) on the Oncotype DX test. Most trials evaluated the test against alternative decision-making strategies; only 2 trials evaluated patient groups, the remaining 42 trials used a mathematical model. Considering the improvement in patient outcomes, multigene assays were found to be cost effective in 90% of the included studies, not exceeding the €40,000 cut-off per earned QALY (Qualitative Adjusted Life-Year).

## INCLUSION OF MULTIGENE TESTS IN CLINICAL GUIDELINES

Due to the strength of data and the clinical utility, the multigene tests have been included in the main international guidelines [42]. The AIOM 2019 guidelines [3] describe the use of Molecular Multigene Prognostic Tumor Molecular Tests (TMMP, Table 5); however, in Italy, these tests are not yet included in the Livelli Essenziali di Assistenza (Essential Assistance Levels, LEA) and therefore are not reimbursed; they are used without specific institutional rules, but on the basis of the clinical needs of individual cases and the possibility for patients to directly cover their cost, with the exception of Lombardy Region, where the 4 tests are reimbursed if used to solve therapeutic questions in patients whose disease characteristics make the choice particularly complex. Therefore, this problem needs to be solved, also in light of the recent document: “Multigene prognostic tests to guide the decision on adjuvant chemotherapy in the treatment of early stage breast cancer”, where the Agenzia Nazionale per i Servizi Sanitari Regionali (AgeNaS) gives a positive opinion on the clinical utility of multigene tests, taking into account the indications and considerations of the EUnetHTA report 2018. The Italian Ministry of Health should therefore take charge of raising the issue of fair access of patients to multigene tests, in order to include them in LEAs with a national reimbursement. All major tests are considered by international guidelines, whose recommendations range from “strong” to “moderate” depending on the quality of evidence and the type of studies (prospective or retrospective).

**Table 5:** Guidelines recommendations on multigene tests for the therapeutic decisions in early breast cancer

Guidelines	Oncotype DX^®^	EndoPredict^®^	Prosigna^®^	MammaPrint^®^
**IQWiG (Germany)** **2020 [43]**	**Yes** (predictive & prognostic) – indicated for: ER+, HER2-, N0 invasive early breast cancer	**No** (the Oncotype DX values cannot be translated to other tests)	**No** (the Oncotype DX values cannot be translated to other tests)	**No** (the Oncotype DX values cannot be translated to other tests)
**NICE (UK)** **2018 [44]**	**Predictive & prognostic** Predictive for: ER+, HER2-, N0 eBC Prognostic for: ER+, HER2-, N0/N1 eBC (pre and post-menopausal)	**Prognostic** indicated for: invasive ER+, HER2-, N0/N1 eBC (pre e post-menopausal)	**Prognostic** indicated for: ER+, HER2-, N0/N1 eBC (post-menopausal only)	**Considered non “cost effective”**
**St. Gallen (EU)** **2019 [45]**	**Specific strong recommendation** for T1–T3 N0 patients (TAILORx cut-offs) Generic strong recommendation for the use of CT in TxN1 patients	**Generic recommendation** (all MGAs are strongly recommended for the use of CT in T1–T3 N0 & TxN1 patients)	**Generic recommendation** (all MGAs are strongly recommended for the use of CT in T1–T3 N0 & TxN1 patients)	**Generic recommendation** (all MGAs are strongly recommended for the use of CT in T1–T3 N0 & TxN1 patients)
**ESMO (EU)** **2019 [46]**	Level of Evidence & Grade of Recommendation (GoR): **1,A** (ER+, HER2-, N0/N1)	Level of Evidence & GoR: **1,B** (ER+, HER2-, N0/N1)	Level of Evidence & GoR: **1,B** (ER+, HER2-, N0/N1)	Level of Evidence & GoR: **1,A** (ER+, HER2-, N0/N1)
**AJCC (US)** **2017 [47]**	**Level 1**	**Level 2**	**Level 2**	**Level 2**
**ASCO (US)** **2019 [29]**	**Strong** Quality of Evidence (EQ): high TAILORx cut-offs (ER/PgR+, HER2-, N0)	**Moderate** EQ: intermediate (ER/PgR+, HER2-, N0)	**Strong** EQ: high (ER/PgR+, HER2-, N0)	**Strong** EQ: high For high risk patients only
**NCCN (US)** **2020 [48]**	Predictive: **Yes** Prognostic: **Yes** NCCN category: **Preferred** Level of Evidence: **1**	Predictive: **No** Prognostic: **Yes** NCCN category: **Other** Level of Evidence: **2A**	Predictive: **No** Prognostic: **Yes** NCCN category: **Other** Level of Evidence: **2A**	Predictive: **No** Prognostic: **Yes** NCCN category: **Other** Level of Evidence: **1**
**AIOM (Italy)** **2019 [3]**	**Prospective validation with RCTs**	**Retrospective validation**	**Retrospective validation**	**Prospective validation with RCTs**
**AgeNaS (Italy)** **2019 [49]**	**Predictive and prognostic value** (ER+, HER2-, N-, N1 for pre and post-menopausal patients)	**Prognostic value** (ER+, HER2-, N-, N1 for pre and post-menopausal patients)	**Valore prognostico** and molecular subtype (for post-menopausal patients only)	**Predictive and prognostic value** with molecular subtype (for stage I/II T1–T2 patients only)

Abbreviations: CT, chemotherapy; eBC, early breast cancer; EQ, Quality of Evidence; ER, hormonal receptor; GoR, Grade of Recommendation; HER2, human epidermal growth factor receptor 2; MGAs, multigene assays; PgR, progesterone receptor; RCT, randomized controlled trial.

## CONCLUSIONS

The benefits of adjuvant chemotherapy in patients with HR+ HER2- early breast cancer should always balanced against its potential short and long-term adverse effects. For this reason, it is essential to identify the appropriate patients where this therapeutic approach can offer the highest clinical benefit.

The clinical and pathological factors are exclusively prognostic and are not able to predict the benefit related to chemotherapy. Consequently, in most cases of luminal disease (ER+/HER2–), the traditional parameters are not sufficient to reliably identify patients who benefit from the hormone therapy alone compared to those for whom a combined hormone-chemotherapy is indicated. Multigene tests, which have demonstrated greater efficacy and reproducibility, can be used for these “intermediate risk” patients. Studies comparing different multigene tests showed that they are not interchangeable, as they provide different and inconsistent results. It is therefore essential to recognize the potential and limitations of each test, in order to choose the best test for the appropriate patient and for the appropriate question.

To date, the Oncotype DX Breast Recurrence Score test is the only validated multigene test, with a IA level of evidence, as actionable test (i.e., suitable to guide therapeutic choice), to support the use of hormone therapy alone, or chemo-endocrine therapy. The PONDx study confirmed that the Oncotype DX test is able to significantly impact therapeutic decisions, reducing the use of chemotherapy (over-treatment) up to 49% and, at the same time, supporting the use of chemotherapy (up to 12%) in potentially under-treated patients. The MammaPrint^®^ test has been validated with prospective evidence supporting its prognostic value, and the actual guidelines only recognize its prognostic validity. Some HTA analysis raised some issues about the cost-effectiveness of the test.

The EndoPredict^®^ and Prosigna^®^ tests are validated only on retrospective trials, although more extensive studies supporting their prognostic value are ongoing. More specifically, EndoPredict^®^ is able to estimate the long-term risk of replase, information particularly useful when the hormone therapy is prolonged beyond 5 years. On the other hand, Prosigna^®^, other than defining the 10-year relapse risk, is also used in research for the analysis of the molecular subtypes of breast cancer.

Due to the level of clinical evidence and the clinical utility, multigene tests have been included in the main international guidelines, with recommendations ranging from “strong” to “moderate” depending on the level of evidence, as well as the characteristics of the different tests.

Based on the high scientific evidence, several European health care systems approved the integration of these tests in the clinical pathway and reimbursement for breast cancer patients. A rapid introduction of multigene tests in the LEAs, in order to allow all Italian women to obtain the same treatment opportunity, is desirable.

## Abbreviations

AgeNaS: Agenzia Nazionale per i Servizi Sanitari Regionali
ASCO: American Society of Clinical Oncology
CI: confidence interval
CT: chemotherapy
DR: distant relapse
eBC: early breast cancer
EBCTCG: Early Breast Cancer Trialists’ Collaborative Group
ED + H: Emergence Department + Hospitalization
EQ: Quality of Evidence
ER: estrogen receptor
ESMO: European Society for Medical Oncology
FDA: Food and Drug Administration
FFPE: Formalin-Fixed Paraffin-Embedded
f-up: follow-up
GoR: Grade of Recommendation
HER2: Human epidermal growth factor receptor 2
HR: Hazard Ratio
HT: Hormone therapy
IQWiG: Institute for Quality and Efficiency in Healthcare
ISTAT: Istituto Nazionale di Statistica
LEA: Livelli Essenziali di Assistenza
LN: lymph node
MGAs: multigene assays
NCCN: National Comprehesive Cancer Network
NCI-CTCAE: National Cancer Institute Common Terminology Criteria for Adverse Events
NGS: Next Generation Sequencing
NICE: National Institute for Health and Care Excellence
NPV: Negative Predictive Value
PPV: Positive Predictive Value
PgR: Progesteron receptor
QALY: Qualitative Adjusted Life-Year
qRT-PCR: quantitative Reverse Transription Polymerase Chain Reaction
RCT: Randomized Controlled Trial
RS: Recurrence Score
RT-PCR: Reverse Transription Polymerase Chain Reaction
TMMP: Test Molecolari Multigenici Prognostici di Tumori
WHO: World Health Organization
